# Editorial: Structure, function and development of neural circuits

**DOI:** 10.3389/fncir.2025.1573101

**Published:** 2025-03-11

**Authors:** Xiangmin Xu

**Affiliations:** ^1^Department of Anatomy and Neurobiology, School of Medicine, University of California, Irvine, Irvine, CA, United States; ^2^Center for Neural Circuit Mapping, School of Medicine, University of California, Irvine, Irvine, CA, United States

**Keywords:** neural circuits, cell types, circuit development, systems neuroscience, developmental disorders, connectivity, viral genetic tool development

Neural circuits are fundamental to the brain's ability to receive sensory inputs, perform computations, and generate appropriate behavioral responses. Investigating neural circuits is a central focus of neuroscience research, as it helps us to understand how the brain processes information, generates behavior, and carries out cognitive functions. The field has made remarkable progress in understanding the nervous system. We predict a substantial acceleration of our understanding of the nervous system, which may foster the development of new therapeutic strategies to treat diseases over the forthcoming decades.

The Research Topic serves as a repository of the research presentations and communications at the 2023 summer conference titled “*Structure, function and development of neural circuits*” (21–23 August 2023), co-sponsored by the Cajal Club, the Allen Institute for Brain Science, and the UC Irvine Center for Neural Circuit Mapping. The main Conference was held at the Beckman Center of the National Academies of Sciences & Engineering near the UC Irvine campus located in the beautiful city of Irvine in Southern California. Additional workshops were held on the UCI campus. In this front-of-the-field conference, leaders and attendees from diverse institutions presented their research progress and technology advancements and brought unique perspectives to the theme of integrating the different facets of neural circuits—structure, function, development, and disease.

This Research Topic welcomed all article types, including research reports, method papers, and reviews and perspectives related to studying the aspects of structure, function, and development of neural circuits in health and disease. All conference participants were encouraged to submit their work to the Research Topic; the Research Topic was also open to non-conference attendees who were interested.

The Research Topic's co-editors included Dr. Christine Gall of the University of California, Irvine, Dr. Sandra Jurado of the Institute of Neurosciences of Spanish National Research Council, Dr. Orkide O Koyuncu of the University of California, Irvine, and Dr. Xiangmin Xu of the University of California, Irvine. We thank all the authors for their efforts leading to a nice Research Topic collection, including the nine manuscripts listed below.

1. Huang et al.
2. Ding et al.
3. Zhu et al.
4. Islam and Blaess
5. Cai et al.
6. Gao et al.
7. Garduño et al.
8. Iqbal et al.
9. Takahata

